# Impact on core values of family medicine from a 2-year Master’s programme in Gezira, Sudan: observational study

**DOI:** 10.1186/s12875-019-1037-1

**Published:** 2019-10-28

**Authors:** Khalid Gaffer Mohamed, Steinar Hunskaar, Samira Hamid Abdelrahman, Elfatih Mohamed Malik

**Affiliations:** 1 Department of Family and Community Medicine, Faculty of Medicine Medina, University of Taibah, Medina, Saudi Arabia; 2 Department of Global Public Health and Primary Care, University of Bergen, Bergen, Norway; 3 Department of Family and Community Medicine, University of Gezira, Medani, Sudan; 4 National Centre for Emergency Primary Health Care, NORCE Norwegian Research Centre, Bergen, Norway; 5 Department of Community Medicine, Faculty of Medicine, University of Khartoum, Khartoum, Sudan

**Keywords:** Attitude, Patient-centred care, General practice, Principles, Sudan, Africa

## Abstract

**Background:**

Training of family physicians should include not only clinical and procedural skills, but also core values as comprehensive care, continuity of care, leadership and patient-centeredness. The Gezira Family Medicine Project (GFMP) is a 2 years Master’s programme in family medicine in Sudan. We assessed the impact of GFMP on the candidates’ adherence to some core values of family medicine.

**Methods:**

This is a prospective study with before-after design based on repeated surveys. We used Patient-Practitioner Orientation Scale (PPOS) to assess physicians’ attitude towards patient-centeredness. Practice based data from individual patients’ consultations and self-assessment methods were used to assess physicians’ adherence to core values.

**Results:**

At the end of the programme the candidates (*N* = 110) were significantly more active in community health promotion (*p* <  0.001), more confident as a team leader (*p* = 0.008), and showed increased adherence to national guidelines for managing diabetes (*p* = 0.017) and hypertension (*p* = 0.003). The responding candidates had more knowledge about patients’ medical history (*p* <  0.001), family history (*p* < 0.001) and family situation (*p* < 0.001). There were more planned follow up consultations (*p* < 0.001) and more referrals (*p* = 0.040). In contrast, results from PPOS showed slightly less orientation towards patient-centeredness (*p* = 0.007).

**Conclusions:**

The GFMP Master’s programme induced a positive change in adherence to several core values of family medicine. The candidates became less patient-centered.

## Background

Since the first university departments of family medicine were established 50–60 years ago, the discipline has steadily developed and is now an integral part of the curricula in the majority of medical schools worldwide [1, 2]. The importance of primary care was stated in the classical Declaration of Alma-Ata in 1978, which is still an important document for politicians and stakeholders [3–5]. However, excessive specialization of health care providers and fragmentation of health care systems still discourage a holistic approach to individuals and their families in most countries. This occurs in spite of the accumulated research evidences and also WHO reports which continuously state that primary care is effective for preventing diseases and reducing deaths, and provides more accessible, equitable and affordable care for the peoples [3–8].

The ideas of family medicine based primary care contains some core values that should guide family physicians not only during the curative work, but also during roles in preventive care, health promotion, community mobilization, research, leadership and resource allocation [5–9]. Many postgraduate family medicine training programmes have emerged worldwide to promote the specialty and its values, so also in low and middle income countries (LMIC) [10–12]. As an example, in Sub-Saharan Africa the Primafamed project has provided a network for collaboration, information, experience exchange and resources sharing for those engaged in such training [10].

While clinical spectrum and practice, necessary equipment, and competence in procedural skills may vary much in different geographical settings [13–15], there are some core principles of family medicine which are unique and universal [9, 13]. Examples of such values include worldwide accepted concepts like patient-centered care, a family perspective, continuity of care, and comprehensive care. “Patient Centred Care” means practitioner’s care about patient’s ideas, concerns, feelings, perspectives and expectations, and also decision sharing. Family medicine has traditionally a strong focus on family perspectives and family background. “Continuity of care” emerges from the presence of a family physician inside the community, it leads to valuable connections over time between patients and their family physician, with positive implications for trust, diagnosis and management, especially for chronic diseases. “Comprehensiveness” is a unique value in family medicine, as family physicians deal with disease entities in all age groups for both sexes, requiring a wide range of competencies.

There is little research in the African context on family physicians’ orientation towards patient-centered medicine or other core values of the specialty, and how such values eventually are practiced. The undergraduate study at the Faculty of Medicine, University of Gezira, is a 5 years programme with a problem based, integrated, and community oriented curriculum. The postgraduate clinical training in Sudan is confined to the Sudanese Medical Specialization Board (SMSB), the only institute which issues the 4–5 years full clinical specialty (MD). Due to the need for family physicians, universities are allowed to train graduated doctors for 2 years in family medicine (Master degree), master candidates are allowed to continue 2 years more to obtain a full family medicine specialty from SMSB. Gezira Family Medicine Project (GFMP) is a 2 years, in-service based Master’s programme in family medicine in Sudan, provided by the Faculty of Medicine, University of Gezira. Detailed descriptions and evaluations of the GFMP and its curriculum and teaching methods have been described in previous articles [16–18].

The aim of the present study is thus to evaluate the impact of the GFMP programme on the candidates’ orientation towards patient-centeredness, their adherence to some core values of family medicine, like team leadership, continuity of care, comprehensive care, health promotion. We also investigated adhesion to the national clinical management guidelines and gender differences.

## Methods

### Study area

Gezira State is situated south to the capital Khartoum. Gezira population is of about 3.7 millions, with mainly rural areas in an agricultural environment. Tropical diseases like malaria are still endemic [19], overshadowing the growing global challenge of non-communicable diseases (NCDs) [19–21].

Primary health care is provided through health centers, which are served by generalist doctors who have a Bachelor degree of Medicine and Surgery, but without any postgraduate training. The number of doctors working in primary care before 2010 was about 115, thus providing a ratio of primary health care doctor to population of 1:32,000. This ratio is far from the ratio proposed by Barbara Starfield; one family doctor for each 1000–1500 inhabitants in order to have a well-functioning health care system [6]. Small rural hospitals represent the second line for referral in Gezira, but more commonly used are the three main city hospitals in Medani, Managil and Kamlin. To bridge the huge lack of primary care doctors, the Gezira Family Medicine Project (GFMP) was planned. The project aims to train doctors in family medicine by means of a 2-year in-service Master’s degree programme.

### Gezira family medicine project (GFMP)

The project was stablished in 2010, as a collaboration project between the Faculty of Medicine at University of Gezira, the State Ministry of Health and other relevant stakeholders [16]. The Faculty of medicine took the responsibility for the candidates’ training in family medicine, while the Ministry of Health provided economic support to the health centers, equipment and staff, including jobs for the enrolled doctors. By providing salaried jobs and a potential achievable academic Master’s certificate, the project was able to recruit 207 physicians to join the programme, and also to provide service in districts that had never been served by doctors before. The majority was new candidates recruited into primary care for the first time. There was, however, a continuous decline in the number of candidates participating during the course, and only 125 were still enrolled at the graduation time. The vast majority of those who did not complete the programme immigrated to other rich countries, reflecting the global trend of brain drain among physicians [22].

The curriculum was designed as a 2 years Master’s programme in family medicine [16]. An in-service model of training was designed, thus allowing the candidates to work clinically at their centers during the programme period. The curriculum constituted of four semesters. The fourth semester was allocated for research and thesis while the first three semesters were divided into modules for the various clinical disciplines like surgery, internal medicine, pediatrics etc. Communication skills including patient-centeredness were taught theoretically at the start of the Master programme within the family medicine module. There was an introductory course in family and community medicine, the aim was to give an orientation about the discipline and its core values. Thereafter the candidates were distributed to their health centers and allocated to the different clinical rotations at hospitals 1 day per week they were allocated to one of the major hospitals to have training in clinical procedural skills, while theoretical teaching was presented as online lectures and case discussions. A continuous evaluation of candidates was performed, checking their knowledge, skills and attitudes. During exams the candidates were asked to show their social accountability and health promotional activities in reports, meetings minutes and pictures, they were also asked to present a map of their catchment area together with demographic data about his/her population, including a chronic disease register.

### Study design and study population

Data were collected at the start of the GFMP in November 2010 “before”, and at the end of the project just before the final exam “after” in 2012/2013. All enrolled candidates were targeted at start of the GFMP (*N* = 207) and at the end (*N* = 125). Only candidates who responded to both the “before” and “after” questionnaires were included when comparing the two groups.

### Data collection

Self-evaluation questionnaires developed for this study were used to assess candidates’ interest in family medicine, confidence in performing certain professional skills and in following national clinical guidelines (Additional file 1 and Additional file 2).

The Patient-Practitioner Orientation Scale (PPOS) [23, 24]. It differentiates between patient-centered approach and doctor-centered approach. It consists of 18 items with two subscales (dimensions), each with nine items. Items are answered using a 6-point Likert-type format. The sharing subscale is reflecting the physician’s attitudes towards sharing information, power and control with the patient, while the caring subscale is reflecting physician’s attitude towards caring about patients’ expectations, feelings and preference. High total scores indicate a more patient-centered attitude, while lower scores indicate a more doctor-centered attitude.

Another questionnaire (Additional file 3) was used to assess whether physicians’ practice is in harmony with some core values of family medicine. Each candidate was expected to collect data from 100 patient consultations during the first programme period and 50 consultations during the last period. Results for each item were calculated as a percentage score for that candidate, with the number of valid patient questionnaires as the denominator. We then present the means of the percentages from all candidates. All questionnaires were anonymous with a coding number. The code number was the same for the pretest (before) and the post-test (after) questionnaires.

### Statistical analysis

SPSS® programme version 23 was used for data management and statistical analyses. Results are presented as descriptive statistics with means, proportions and percentages. “Paired sample t tests” was used to test the significance of quantitative data, while McNemar-Bowker test was used to test differences in categorical data.

## Results

Of the 207 candidates who were enrolled in the GFMP at the start, 188 candidates (91%) responded to the “before” questionnaire. At the end of the Master programme 115 (92%) of the targeted 125 candidates still in the programme responded to the “after” questionnaire, of whom 110 answered both the “before” and “after” surveys.

### Interest in family medicine and progress in certain professional skills

Table 1 shows how the candidates evaluated their own interest in the specialty of family medicine and their self-assessment for competence in some professional roles as a family physician. There were no statistically significant differences at the start between all candidates and those included in the before-after analyses. The candidates showed generally a high interest in family medicine specialty at the start, and a significantly increased interest at the end. The category of “very much interest” had increased by 56% for males and 78% for females. However, more males were “very much interested” than females both “before” and “after”, but the differences were not statistically significant (48% vs. 32%, *p* = 0.19 and 74% vs. 58%, *p* = 0.20).

**Table 1:** Physician’s self-evaluation regarding interest in family medicine and certain professional skills

Question	All (*N* = 188)	All (*N* = 110)			Males (*N* = 43)			Females (*N* = 67)		
		Before	After	*P* value	Before	After	*P* value	Before	After	*P* value
Physician’s interest in family medicine										
Not/Little/Somewhat	23.4	16.8	7.3	< 0.001	16.7	7.0	0.022	16.9	7.6	0.002
Much	43.1	44.9	28.4		35.7	18.6		50.8	34.8	
Very much	33.5	38.3	64.2		47.6	74.4		32.3	57.6	
Self-evaluation regarding caring about patient’s psychosocial aspects										
Yes	45.7	49.5	74.8	0.001	48.8	73.8	0.056	50.0	75.4	0.001
Sometimes	42.5	39.0	17.8		36.6	23.8		40.6	13.8	
No	11.8	11.4	7.5		14.6	2.4		9.4	10.8	
Practicing health promotion at the community level										
Yes	17.3	21.5	82.5	< 0.001	33.3	86.2	< 0.001	15.0	80.4	< 0.001
Sometimes	3.2	2.2	2.5		3.0	3.4		1.7	2.0	
No	79.5	76.3	15.0		63.6	10.3		83.3	17.6	
Physicians’ satisfaction regarding the communication with local community										
Not/Little/Somewhat	44.9	38.8	24.8	< 0.001	26.8	11.6	0.077	46.8	33.3	0.001
Much	39.3	42.7	29.4		39.0	27.9		45.2	30.3	
Very much	15.8	18.4	45.9		34.1	60.5		8.1	36.4	
Confidence as a health team leader										
Very confident	16.8	19.2	35.5	0.008	26.8	41.9	0.378	14.3	31.3	0.018
Confident	54.1	53.8	52.7		48.8	48.8		57.1	55.2	
Not fully confident/Uncertain/Not able	29.1	26.9	11.8		24.4	9.3		28.6	13.4	
Physicians’ satisfaction regarding the communication with other employees										
Not/Little/Somewhat	36.1	36.9	16.5	< 0.001	26.8	14.0	0.015	43.5	18.2	< 0.001
Much	41.5	43.7	33.0		51.2	34.9		38.7	31.8	
Very much	22.4	19.4	50.5		22.0	51.2		17.7	50.0	

Data compares before and after GFMP by gender including candidates who answered both before and after questionnaires (*N* = 110) and from all who participated in the before questionnaire (*N* = 188). All numbers are column distribution percentages. *P*-values from McNemar-Bowker tests

Large and statistically significant improvements were also found in certain professional role skills, like health team leadership, communication with colleagues, communication with the local community, practicing health promotion, and caring about patients’ psychosocial aspects (Table 1). For males the increase did not reach statistical significance for all questions, but the changes were at the same magnitudes as for females, except for communication with the local community, where males scored higher than females both before (*p* < 0.01) and after (*p* < 0.05).

Candidates showed a very high adherence to malaria guidelines both before and after the programme, with no statistically significant change and small gender differences. For diabetes mellitus and hypertension, we found statistically significant increases in adherence for the total group, the increase was statistically significant for females but not for males (Table 2).

**Table 2:** Candidates’ self-evaluation regarding adherence to the national clinical guidelines

National guidelines topic	All (*N* = 110)			Males (*N* = 43)			Females (*N* = 67)		
	Before	After	*P* value	Before	After	*P* value	Before	After	*P* value
Malaria									
Yes	83.0	88.1	0.259	78.0	92.9	0.142	86.2	85.1	0.362
Sometimes	13.2	7.3		17.1	4.8		10.8	9.0	
No	3.8	4.6		4.9	2.4		3.1	6.0	
Diabetes mellitus									
Yes	50.0	65.5	0.017	52.4	65.1	0.382	48.4	65.7	0.020
Sometimes	21.7	24.5		23.8	23.3		20.3	25.4	
No	28.3	10.0		23.8	11.6		31.3	9.0	
Hypertension									
Yes	49.5	67.3	0.003	48.8	60.5	0.133	50.0	71.6	0.013
Sometimes	18.7	20.0		17.1	27.9		19.7	14.9	
No	31.8	12.7		34.1	11.6		30.3	13.4	

It includes malaria, diabetes mellitus, and hypertension before and after GFMP master programme (*N* = 110), by gender. All numbers are column distribution percentages. *P*-values from McNemar-Bowker tests

### Patient-practitioner orientation scale (PPOS) for patient centeredness

A total of 188 of the 207 available candidates answered the PPOS questionnaire at the start of the Master programme (91% response rate), while 108 of the 125 remaining candidates responded to the “after” questionnaire (86% response rate). Only results from candidates who responded both the “before” and “after” questionnaires are included (*N* = 103).

Table 3 presents PPOS mean values for total scores and subscales, standard deviations and *P* values by gender, for results before and after the Master programme. The PPOS scores revealed a small, but statistically significant decrease of 4% in the overall score, mostly due to a decrease of 7% in the sharing subscale. For males, there was a decrease in all scores, while for females we found an increase in the caring subscale, although not statistically significant. Females had higher overall scoring than males, both in the “before” and “after” survey, but the differences were not statistically significant (*p* = 0.210 and *p* = 0.753).

**Table 3:** Patient-Practitioner Orientation Scale (PPOS) scores before and after GFMP master programme distributed by gender

	PPOS score				Change	*P*-value
	Before		After			
	Mean	SD	Mean	SD		
All (*n* = 103)						
Total PPOS score	3.75	0.48	3.60	0.60	−0.15	0.007
Caring subscale	3.37	0.63	3.36	0.73	−0.01	0.950
Sharing subscale	4.14	0.56	3.85	0.60	−0.29	< 0.001
Males (*n* = 41)						
Total PPOS score	3.74	0.44	3.50	0.66	− 0.24	0.011
Caring subscale	3.43	0.62	3.20	0.77	−0.23	0.062
Sharing subscale	4.05	0.51	3.82	0.65	−0.23	0.042
Females (*n* = 62)						
Total PPOS score	3.76	0.50	3.66	0.55	−0.10	0.182
Caring subscale	3.33	0.64	3.47	0.68	0.14	0.120
Sharing subscale	4.20	0.59	3.87	0.58	−0.33	< 0.002

*P*-values calculated by paired t tests

### Core values during clinical consultations

Of the 207 original candidates, 151 delivered patient based data (73%). At the end 116 out of 125 (93%) delivered data, and 91 delivered data for before and after comparisons.

The candidates had large and statistically significant improvements in all investigated family medicine core value topics, such as continuity of care and knowledge of the patients’ family and medical history (Table 4). There was a shift in consultations from first contacts to more planned controls and follow-ups. The referral rate increased from 14% to about 17%.

**Table 4:** Family medicine core values in relation to candidates’ practice before and after GFMP

Core value topic	All (*N* = 91)			Males (*N* = 40)			Females (*N* = 51)		
	Before	After	*P* value	Before	After	*P* value	Before	After	*P* value
Patients presenting as a first contact regarding the main contact reason	78.5	62.7	< 0.001	77.57	60.6	< 0.001	79.3	64.4	< 0.001
Patients whom the doctor is the personal doctor for the patient	34.7	74.6	< 0.001	30.4	75.9	< 0.001	38.0	73.5	< 0.001
Patients with planned control in the future for follow up	47.6	61.0	< 0.001	48.7	60.3	0.054	46.8	61.4	0.002
Patients who are referred	13.7	17.3	0.040	10.4	17.3	0.009	16.2	17.2	0.678
Patients whom the doctor knows their medical history	56.2	87.0	< 0.001	44.3	85.3	< 0.001	65.6	88.3	< 0.001
Patients whom the doctor knows their family situation.	49.4	84.0	< 0.001	41.0	84.7	< 0.001	56.7	83.5	< 0.001
Patients whom the doctor knows important parts of their family medical history	47.3	84.0	< 0.001	34.4	82.3	< 0.001	57.1	85.2	< 0.001

Data based on questionnaires filled by the candidates during individual patients’ consultations and presented as mean of the candidates’ percentages of their own patients. *P*-values by paired t test

## Discussion

The candidates of this family medicine Master’s programme in Gezira, Sudan, showed progress in most topics related to core values and competencies, including leadership, continuity of care, comprehensive care, and adherence to national clinical guidelines. However, the study revealed a reduction in candidates’ self-assessed patient-centeredness.

### Family medicine interest and role aspects

Not surprisingly, the enrolled candidates expressed a high interest in family medicine even at the start of the GFMP. However, at the end the group with “very much” interest had increased both for males and females. The GFMP was thus able to recruit a large group of motivated students, and to increase their motivation and interest during the programme. This is consistent with an Egyptian study, which showed increased interest in family medicine as a career after an orientation course in family medicine for house-officers [25].

The substantial increase in the candidates’ participation in community health promotion may be explained by the high emphasis on this issue through the curriculum, by claiming pictures, reports, and meeting minutes as documentation at exams. We also observed significant improvements in competencies like leadership, communication with the local community, and communication with the other employees. Males self-evaluated themselves as more confident in leadership than females, while females reported larger progress.

### Clinical guidelines

The self-evaluation for adherence to the national clinical guidelines concerning malaria, diabetes mellitus, and hypertension, showed high adherence already at the beginning. Many candidates obviously knew the guidelines from before. There was a significant increase in adherence for diabetes and hypertension, although the malaria guidelines still had the highest score at the end. The high score is most probably due to the high local and international emphasis on “rolling back” malaria [26]. Guidelines for hypertension and diabetes are not so widely distributed or advocated, and candidates might have followed guidelines and good practice even if they did not express recognition of national guidelines.

On the other hand, there are many studies concluding that guidelines are not adequately implemented, and that physicians’ knowledge of guidelines in itself does not lead to better guideline implementation [27, 28]. In family medicine, there has also been a focus on negative consequences when obliged to apply a variety of single disease guidelines to multi-morbid patients, including increased risk of poly-pharmacy and overtreatment [29]. We have no data showing the actual compliance with the guidelines on an individual patient level. A prerequisite for use in clinical practice, however, is knowledge of their existence and a statement of adherence.

### Patient centeredness

To our knowledge, this is the first prospective study measuring physician’s attitude towards patient-centeredness in an African context. The results for the whole cohort showed a slight, but statistically significant reduction in the total score from 3.75 to 3.60, while the aim was to increase the level. Compared with results from other countries, the total PPOS score in our study is higher than found among Pakistani students (score 3.40) [30], but lower than 4th year US medical students (score 4.46) [31], Brazilian students (score 4.66) [32] and Greek 6th year medical students (score 3.81) [33]. Cross-cultural variations are found also within the same country, as a US study using PPOS revealed significant differences between medical students of different ethnicity [31].

A decrease in PPOS scores after clinical rotations is found in other studies when comparing pre-clinical and post-clinical clerkship students [31, 34, 35], although the results are heterogeneous [30, 32, 36]. A decline is usually explained by the shift from the ideal theoretical teaching in pre-clinical years towards more biological teaching in hospital settings. This explanation may be plausible in our setting. Our candidates had a course in communication skills at the very beginning, and the PPOS questionnaire was distributed after this course. Our candidates had a theoretical course in communication skills and patient-centeredness at the very beginning of the Master program, and the PPOS questionnaire was distributed after this course, contributing to a high score. In contrast, we assume that the clinical clerkships at hospitals concentrated more on the clinical skills and biological aspects and less on patient-centeredness and communication skills.

Our scores in the sharing subscale are lower than in the caring subscale; this is consistent with other studies [24, 31]. If valid, the findings mean less involvement of patients in information and decision-making. This might be a result of the dominating paternalistic biomedical model in medical education and practice, well recognized also in our settings. Strategies to address this challenge include formal courses and further emphasis of patient-centeredness issue both in undergraduate and postgraduate curricula, in pre-clinical and clinical clerkships. Training of trainers (TOT) is important for introducing issues like the possibility and benefits of implementing and applying patient-centeredness even in our high disease burdened settings. Female gender is traditionally associated with more patient-centeredness [31, 33, 37]. Although females scored higher than males in our study, the differences were not statistically significant, consistent with studies from Pakistan [30] and Nepal [38].

### Core values from clinical practice

The reduced number of first contacts and increased number of patients with planned follow-up reflect a practice of more continuous care, but may also reflect patients’ satisfaction with and confidence in their family doctor. Increased knowledge of patients’ medical history, family medical history, and patients’ family situation, is an indication of improved patient-physician relationship. Unexpectedly, the referral rate was increased, mainly among the male candidates. It may be assumed that the referral rate may decline if the physicians developed more clinical procedural skills, as described in a previous paper [16]. However, it can also indicate a shift towards patients with more chronic diseases and multi-morbidity, best managed in primary care but with some need of specialist investigations and evaluation. Another explanation is better and more communication and collaboration between the family medicine candidates and the hospital doctors, as a results of clinical rotations at hospitals [16] and telemedicine interaction [17]. The referral rate of 17% is still within the normal expected rate internationally [39], as Starfield assumed that between 15 and 25% of population needs referral from primary care to secondary or tertiary care [40].

### Study limitations

Self-assessment is a subjective method, and may reflect candidates’ confidence rather than competence [41, 42]. Self-reporting can result in social desirability bias, it occurs when respondents try to give acceptable results for investigators. We cannot exclude such bias induced by our methods. However, this method is widely accepted, used and easy as an assessment indicator for curriculum development [43, 44]. The drop out of candidates during the course of the Master’s programme from 207 to 125 is a study limitation, regarding both representability and statistical power. However, administrative data show that more than 80% left to rich countries due to economic reasons (GFMP, personal communication). In a previous work [18] we compared clinical skills between candidates who participated before the drop out with the group of candidates who continued to the end of the master program. Results showed 1.2% difference in mean clinical skills’ confidence between the two groups, compared with a difference of 21.7% in clinical skills before and again after the aster program. That indicates that the drop out group was not different to the rest in this respect.

The use of PPOS for measuring physician-patient relationship has a number of challenges, including cross cultural variability, reliability and validity [31]. We do not know if and how national or local societal cultures or educational and teaching practices in Africa could have had impact on the results. There are other validated tools available [45]. Other tools could have been used in assessing patient-centeredness, but the problem of a proper validation in the African context will still be there. We decided to use the PPDS since it has been used in different settings and cultures. The PPOS explores the physician’s attitude, but not the physician’s practice (behavior), reminding us again about the questionable link between attitude and actual behavior. Strategies to foster patient centeredness in medical curricula are needed, and may need research and development in an African context. The theoretical component in the pre-clinical years needs to be followed by a role model of patient-centered practice in the clinical years. Tools like simulated patients and video taping of consultations might help in training. Questionnaire based feedback from patients might help in assessing and developing patient-centeredness in post graduate training.

Although the presented data is dated 2010–2012, we assume it is still of valuable benefit since the Master curriculum is still on use with minimal changes, and the results are of high interest for family medicine training programmes. The results may guide other universities in Africa and add to the literature about postgraduate teaching of family medicine in Africa.

## Conclusions

The GFMP Master’s programme had a significant positive impact on the candidates’ adherence to family medicine core values like leadership, continuity of care, comprehensive care, and compliance with national clinical guidelines. However, the study revealed a reduction in candidates’ self-assessed patient-centeredness.

### Practical implication and further research

Family medicine training programmes in sub-Saharan Africa and globally are progressing quickly, and results from scientific evaluations are important for sharing experiences and curriculum development. GFMP is now a well known example of how to scale up family medicine in Africa, and it has inspired other countries in the region [46]. Future curricula should aim to train and assess family physicians in core values as well as clinical work and procedural skills. Patient-centeredness is an area that needs focus, including further studies both at undergraduate and postgraduate level. Further research is needed to develop new assessment tools which are more valid and reliable in the African context That includes tools to assess patient-centeredness, leadership, and also other family medicine core values.

## Supplementary information

**Additional file 1.** Student’s questionnaire before GFMP.

**Additional file 2.** Student’s questionnaire after GFMP.

**Additional file 3.** Patient’s questionnaire.

## Data Availability

The datasets used and/or analyzed during the current study are available from the corresponding author on reasonable request.

## Abbreviations

GFMP: Gezira Family Medicine Project
ICT: Information and Communication Technology
LMIC: Low and Middle Income Countries
NCDs: Non-Communicable Diseases
PPOS: Patient-Physician Orientation Scale
WHO: World Health Organization
