# Supplementary material for: Impact on core values of family medicine from a 2-year Master’s programme in Gezira, Sudan: observational study
Source: BMC Fam Pract. 2019 Oct 28;20:145. doi: 10.1186/s12875-019-1037-1 (PMC6816210; doi:10.1186/s12875-019-1037-1)
Supplement: Supplementary file 1 — Additional file 1. Student’s questionnaire before GFMP. [file 12875_2019_1037_MOESM1_ESM.docx]

**Doctors number:………….**

**Locality:…………….**

**Please fill this page according to your situation before starting at the Gezira family medicine project:**

1. Age: …………..(Years)
2. Sex: Male□ Female □
3. Year of graduation: ……………… .
4. University of graduation: ………………………………………………….. .
5. How long have you been working in general practice?..................... years.
6. How many hours do you use for academic reading per week?................ hours?.
7. Why did you choose the specialty of family medicine?

Economy □ Easy specialty □ Short time□ Only available opportunity □, Others:

1. Do you have age register? Yes □ No□
2. Do you have sex register? Yes □ No □
3. Do you have a disease register (List of patients with specific diseases) ? Yes □ No □
4. Do you make notes for every consultation which are available for next time? Yes □ No □
5. Do you have comprehensive medical files system for documentation of all medical activities (Notes, letter, lab x-ray results..) ? Yes □ No □
6. Do you know exactly what is you catchment area?

Yes □ No □

1. Do you have Maternal death register?

Yes □ No □

1. Do you participate in the periodic health check up for children-school? Yes □ No □
2. How do you feel confident as a health team leader in your health center? Very confident □

Confident □ Not fully confident □ Uncertain □ Not able □

1. I use to show diabetes patients how to use Insulin injections themselves.

Yes □ No □ Some times □

1. I use to show women how to examine their breasts for ca. breast. Yes □ No □ Some times □
2. I use to discuss with smoking patients the bad effects of cigarette smoking.

Yes □ No □ Some times □

1. I use to advice mothers who have children with malnutrition how to feed them.

Yes □ No□ Some times □

1. Do you have regular meetings with the community to encourage them to participate in health services?
2. Do you practice rehabilitation role for patients with special needs like psychiatry patients?

Yes □ No □ Some times □

1. Do you usually think about the psychosocial aspects of your patients? Yes □ No □ Some times □
2. Do you follow the national guidelines for Malaria management in your patient management?

Yes□ No□ Some times □

1. Do you follow the national guidelines for diabetes management in your patient management?

Yes □ No□ Some times □

1. Do you follow the national guidelines for Hypertension management in your patient management?

Yes □ No □ Some times □

1. **Please fill inn values for all days in a typical week – use the number you think is close to the average**

| Day | **Day working hours.** | | **Number of consultations during the day.** | **Evening working hours** | | **Number of consultations during the evening** | **Number of consultations during the night. (After 22:00)** | **Number of home visit** | |
| --- | --- | --- | --- | --- | --- | --- | --- | --- | --- |
|  | **From** | **To** |  | **From** | **To** |  |  | **Day** | **Night** |
| **Sunday** |  |  |  |  |  |  |  |  |  |
| **Monday** |  |  |  |  |  |  |  |  |  |
| **Tuesday** |  |  |  |  |  |  |  |  |  |
| **Wednesday** |  |  |  |  |  |  |  |  |  |
| **Thursday** |  |  |  |  |  |  |  |  |  |
| **Friday** |  |  |  |  |  |  |  |  |  |
| **Saturday** |  |  |  |  |  |  |  |  |  |

**For the next table, fill in a X for the appropriate category of your opinion**

|  | **Very much** | **Much** | **Somewhat** | **Little** | **Not** |
| --- | --- | --- | --- | --- | --- |
| 1. Are you interested in family medicine specialty? |  |  |  |  |  |
| 1. Are you satisfied regarding the communication with the local community? |  |  |  |  |  |
| 1. Are you satisfied regarding the communication with the other employer in your center |  |  |  |  |  |
| 1. Are you satisfied regarding your income? |  |  |  |  |  |
