# Supplementary material for: Impact on core values of family medicine from a 2-year Master’s programme in Gezira, Sudan: observational study
Source: BMC Fam Pract. 2019 Oct 28;20:145. doi: 10.1186/s12875-019-1037-1 (PMC6816210; doi:10.1186/s12875-019-1037-1)
Supplement: Supplementary file 2 — Additional file 2. Student’s questionnaire after GFMP. [file 12875_2019_1037_MOESM2_ESM.docx]

**Doctors number:………….**

**Locality:…………….**

**Please fill this page according to your situation As it is now:**

1. Do you have age register? Yes □ No□
2. Do you have sex register? Yes □ No □
3. Do you have a disease register (List of patients with specific diseases) ? Yes □ No □
4. Do you make notes for every consultation which are available for next time? Yes □ No □
5. Do you have comprehensive medical files system for documentation of all medical activities (Notes, letter, lab x-ray results..) ? Yes □ No □
6. Do you know exactly what is you catchment area?

Yes □ No □

1. Do you have Maternal death register?

Yes □ No □

1. Do you participate in the periodic health check up for children-school? Yes □ No □
2. How do you feel confident as a health team leader in your health center? Very confident □

Confident □ Not fully confident □ Uncertain □ Not able □

1. I use to show diabetes patients how to use Insulin injections themselves.

Yes □ No □ Some times □

1. I use to show women how to examine their breasts for ca. breast. Yes □ No □ Some times □
2. I use to discuss with smoking patients the bad effects of cigarette smoking.

Yes □ No □ Some times □

1. I use to advice mothers who have children with malnutrition how to feed them.

Yes □ No□ Some times □

1. Do you have regular meetings with the community to encourage them to participate in health services?
2. Do you practice rehabilitation role for patients with special needs like psychiatry patients?

Yes □ No □ Some times □

1. Do you usually think about the psychosocial aspects of your patients? Yes □ No □ Some times □
2. Do you follow the national guidelines for Malaria management in your patient management?

Yes□ No□ Some times □

1. Do you follow the national guidelines for diabetes management in your patient management?

Yes □ No□ Some times □

1. Do you follow the national guidelines for Hypertension management in your patient management?

Yes □ No □ Some times □

1. How many hours do you use for academic reading per week?................ hours?.
2. Why did you choose the specialty of family medicine?

Economy □ Easy specialty □ Short time□ Only available opportunity □, Others:

1. **Please fill inn values for all days in a typical week – use the number you think is close to the average**

| Day | **Day working hours.** | | **Number of consultations during the day.** | **Evening working hours** | | **Number of consultations during the evening** | **Number of consultations during the night. (After 22:00)** | **Number of home visit** | |
| --- | --- | --- | --- | --- | --- | --- | --- | --- | --- |
|  | **From** | **To** |  | **From** | **To** |  |  | **Day** | **Night** |
| **Sunday** |  |  |  |  |  |  |  |  |  |
| **Monday** |  |  |  |  |  |  |  |  |  |
| **Tuesday** |  |  |  |  |  |  |  |  |  |
| **Wednesday** |  |  |  |  |  |  |  |  |  |
| **Thursday** |  |  |  |  |  |  |  |  |  |
| **Friday** |  |  |  |  |  |  |  |  |  |
| **Saturday** |  |  |  |  |  |  |  |  |  |

**For the next table, fill in a X for the appropriate category of your opinion**

|  | **Very much** | **Much** | **Somewhat** | **Little** | **Not** |
| --- | --- | --- | --- | --- | --- |
| 1. Are you interested in family medicine specialty? |  |  |  |  |  |
| 1. Are you satisfied regarding the communication with the local community? |  |  |  |  |  |
| 1. Are you satisfied regarding the communication with the other employer in your center |  |  |  |  |  |
| 1. Are you satisfied regarding your income? |  |  |  |  |  |

***How do you evaluate your ability to* perform the following *skills? (Mark X)***

| No | Question | Very confident | Confident | Not fully confident | Uncertain | Not able |
| --- | --- | --- | --- | --- | --- | --- |
| 1 | Measuring blood pressure |  |  |  |  |  |
| 2 | Hemoglobin measuring |  |  |  |  |  |
| 3 | Urine analysis |  |  |  |  |  |
| 4 | Glucose measuring. |  |  |  |  |  |
| 5 | Blood film for malaria |  |  |  |  |  |
| 6 | Insertion of nasogastric tube |  |  |  |  |  |
| 7 | ECG taking. |  |  |  |  |  |
| 8 | ECG interpretation |  |  |  |  |  |
| 9 | Inhaler technique |  |  |  |  |  |
| 10 | Vein puncture and drip start |  |  |  |  |  |
| 11 | Cardiopulmonary resuscitation. |  |  |  |  |  |
| 12 | Chest X ray in infections. |  |  |  |  |  |
| 13 | Vaginal examination. |  |  |  |  |  |
| 14 | Taking cervical smear |  |  |  |  |  |
| 15 | Evacuation after abortion |  |  |  |  |  |
| 16 | Normal delivery. |  |  |  |  |  |
| 17 | Caesarian section. |  |  |  |  |  |
| 18 | IUCD insertion. |  |  |  |  |  |
| 19 | Abscess drainage. |  |  |  |  |  |
| 20 | Suturing of wounds. |  |  |  |  |  |
| 21 | Urethral catheterization . |  |  |  |  |  |
| 22 | Knee examination |  |  |  |  |  |
| 23 | Insulin treatment in hyperglycaemia |  |  |  |  |  |
| 24 | X-Ray interpretation in trauma. |  |  |  |  |  |
| 25 | Stopping epistaxis |  |  |  |  |  |
| 26 | Eye fundoscopy |  |  |  |  |  |
| 27 | Visual acuity. |  |  |  |  |  |
| 28 | Appendectomy operation. |  |  |  |  |  |
| 29 | Caesarian section. |  |  |  |  |  |
| 30 | Acute abdomen operation. |  |  |  |  |  |
| 31 | Management of diabetic coma |  |  |  |  |  |
| 32 | Management of asthma |  |  |  |  |  |
| 33 | Management of myocardial infarction. |  |  |  |  |  |
| 34 | Management of tuberculosis |  |  |  |  |  |
| 35 | Cholesteatoma operation |  |  |  |  |  |
| 36 | Acute vaginal bleeding |  |  |  |  |  |
| 37 | Management of iIridocyclitis |  |  |  |  |  |
| 38 | Malnutrition in children. |  |  |  |  |  |
| 39 | Tonsillitis. |  |  |  |  |  |
| 40 | Acute psychosis |  |  |  |  |  |
| 41 | Management of major depression |  |  |  |  |  |
| 42 | Removal of foreign body from the eye |  |  |  |  |  |
| 43 | Management of urinary retention |  |  |  |  |  |
| 44 | Diagnosis of hearing loss |  |  |  |  |  |
| 45 | Plaster of minor fractures |  |  |  |  |  |
| 46 | Stabilization of major fractures. |  |  |  |  |  |
| 46 | Thoracal drainage. |  |  |  |  |  |

**Gezira Family Medicine Project**

Fill in a X for each item/question in the appropriate box of agreement

|  | **ITEM** | **Strongly agree** | **Somewhat agree** | **Agree** | **Disagree** | **Somewhat disagree** | **Strongly disagree** |
| --- | --- | --- | --- | --- | --- | --- | --- |
| 1 | The doctor is the one who should decide what gets talked about during a visit |  |  |  |  |  |  |
| 2 | Although health care is less personal these days, this is a small price to pay for medical advances. |  |  |  |  |  |  |
| 3 | The most important part of the standard medical visit is the physical examination. |  |  |  |  |  |  |
| 4 | It is often best for patients if they do not have a full explanation of their medical condition. |  |  |  |  |  |  |
| 5 | Patients should rely on their doctors’ knowledge and not try to find out about their conditions on their own. |  |  |  |  |  |  |
| 6 | When doctors ask a lot of questions about a patient's background, they are prying too much into personal matters |  |  |  |  |  |  |
| 7 | If doctors are truly good at diagnosis and treatment, the way they relate to patients is not that important. |  |  |  |  |  |  |
| 8 | Many patients continue asking questions even though they are not learning anything new. |  |  |  |  |  |  |
| 9 | Patients should be treated as if they were partners with the doctor, equal in power and status. |  |  |  |  |  |  |
| 10 | Patients generally want reassurance rather than information about their health. |  |  |  |  |  |  |
| 11 | If a doctor's primary tools are being open and warm, the doctor will not have a lot of success. |  |  |  |  |  |  |
| 12 | When patients disagree with their doctor, this is a sign that the doctor does not have the patient's respect and trust. |  |  |  |  |  |  |
| 13 | A treatment plan cannot succeed if it is in conflict with a patient's lifestyle or values. |  |  |  |  |  |  |
| 14 | Most patients want to get in and out of the doctor's office as quickly as possible |  |  |  |  |  |  |
| 15 | The patient must always be aware that the doctor is in charge. |  |  |  |  |  |  |
| 16 | It is not that important to know a patient's culture and background in order to treat the person's illness |  |  |  |  |  |  |
| 17 | Humour is a major ingredient in the doctor's treatment of the patient. |  |  |  |  |  |  |
| 18 | When patients look up medical information on their own, this usually confuses more than it helps. |  |  |  |  |  |  |
|  | **ITEM** | **Strongly agree** | **Somewhat agree** | **Agree** | **Disagree** | **Somewhat disagree** | **Strongly disagree** |
| 1 | Telemedicine is of high benefit for my patients |  |  |  |  |  |  |
| 2 | It is a good teaching tool for family doctors |  |  |  |  |  |  |
| 3 | It is the only way of communication with hospital specialists |  |  |  |  |  |  |
| 4 | I used it for the follow up of my patients in more than one consultation |  |  |  |  |  |  |
| 5 | I use it usually when the patient is in my office |  |  |  |  |  |  |
| 6 | My patients are usually satisfied with telemedicine |  |  |  |  |  |  |
| 7 | The patients miss the confidence on family doctor when telemedicine is used |  |  |  |  |  |  |
| 8 | I use the program to get second opinion from other family medicine colleagues |  |  |  |  |  |  |
| 9 | The electronic filing system is highly important for my practice |  |  |  |  |  |  |
| 10 | The patients are not happy when I write on the computer during consultation |  |  |  |  |  |  |
| 11 | I register all my patients on the electronic filing system |  |  |  |  |  |  |
| 12 | One line lectures are a good teaching method |  |  |  |  |  |  |
| 13 | The training at hospital was closely related to our needs in practice |  |  |  |  |  |  |
| 14 | It was easy to combine the work with the training activities |  |  |  |  |  |  |
| 15 | I am generally satisfied with the training program during the master period |  |  |  |  |  |  |
| 16 | I am planning to continue the family medicine speciality to get the MD or PhD |  |  |  |  |  |  |
| 17 | I recommend that the idea of the Gezira Family Medicine Project should be replicated in other places |  |  |  |  |  |  |
| 18 | I am planning to leave Sudan shortly after finishing the master program |  |  |  |  |  |  |
