# Supplementary material for: Impact on core values of family medicine from a 2-year Master’s programme in Gezira, Sudan: observational study
Source: BMC Fam Pract. 2019 Oct 28;20:145. doi: 10.1186/s12875-019-1037-1 (PMC6816210; doi:10.1186/s12875-019-1037-1)
Supplement: Supplementary file 3 — Additional file 3. Patient’s questionnaire. [file 12875_2019_1037_MOESM3_ESM.doc]

**Doctor’s no: ______ Patient no:______**

**Patient’s:**

Age: ____years Sex: □ Male □Female

Cohabitation: □ Married □ Single

**Day of consultation:** _____day (Sun-Sat) **Time of consultation:** _____ hour (00-23)

**Reason(s) for encounter this consultation**

Symptom(s): 1_______________________

2_______________________

3_______________________

Known diagnosis: 1_______________________

2_______________________

3_______________________

**Today’s contact**

□ Patient initiated control for chronic disease

□ Scheduled revisit for known disease/symptom

□ First contact for main symptom

□ Second □Third □Fourth □More

If first contact; how long has the symptom lasted before the consultation: ______ days

**In the past**

Before consultation, has the patient for the new/main problem:

Used self management? □ Yes □ No

if yes: □ life style change □ drugs □ other:

Visited:

□ Local healer □ Other primary care doctor

□ Other primary care worker □ Specialist

Has the patient visited you before? □ Yes □No

Are you the personal doctor for this

patient over time? □Yes □No

Do you for this patient know

- the patient’s medical history? □Yes □No

- the patient’s family situation? □Yes □No

- important parts of the family’s

medical history? □Yes □No

**Diagnosis and use of equipment**

Clinical examination:

□None □Full clinical Partly, which organ:_______________

Lab. investigations requested:

□Hb □Glucose □BFFM

□TWBC □S-creatinin □S-cholesterol

□Urine general □Others:

**Equipment/procedure(s) used/done today**:

1_______________________________

2_______________________________

3_______________________________

**Management/therapy**

Was the patient given therapeutic procedures or any treatment in the room during the consultation? □ Yes □ No

If YES, what:

**Prescriptions**: Write down medication(s) and circle the kind of use

(A)New, short (B)New chronic (C) Renewal

1_____________________________ A B C

2_____________________________ A B C

3_____________________________ A B C

4_____________________________ A B C

**Final diagnosis of main problem today**

1. ___________________________
2. ___________________________

**Follow up**

Was the patient referred?

□ No □ Hospital

□ Radiology unit □ Private doctor/clinic

□ Other:

Planned control in this center:

□ Not needed □ Within 1 week

□ Within 1 month □ More than a month.

**Evaluation of the consultation**

How long did the consultation last?

□ 0-5 min □ 5-10 min

□ 10-15 min □ 20-25 min

□ 20-25 min □ > 25 min; how long:…….

Do you think the patient have payment difficulties for medical problems?

□ Yes, for today’s consultations& lab.

□ Yes, for hospital stay fees.

□ Yes, for X-ray or lab

□ Yes, for medications.

Is the patient a member of the health insurance fund? □ Yes □ No

Overall severity of problem today (mark one):

□ Not at all a serious problem

□ Not very serious, low impact on health

□ Somewhat serious

□ Serious/high impact on health

□ Very serious, potential life threatening
